# Supplementary material for: Fungi in soil and understory have coupled distribution patterns
Source: PeerJ. 2021 Sep 21;9:e11915. doi: 10.7717/peerj.11915 (PMC8462376; doi:10.7717/peerj.11915)

Fungi in soil and understory have coupled distribution patterns

André Boraks, Anthony S. Amend

PeerJ,2021

Supplemental Figure S2

Vanuatu map. Map of sampling location in Vanuatu with transect locations indicated by red squares on map (a). Numbers superimposed on the transect location are Transect ID. Each transect (b) had 36 sampling locations (10 meters by 40 meters), indicated by the red circles on the illustrated transect.

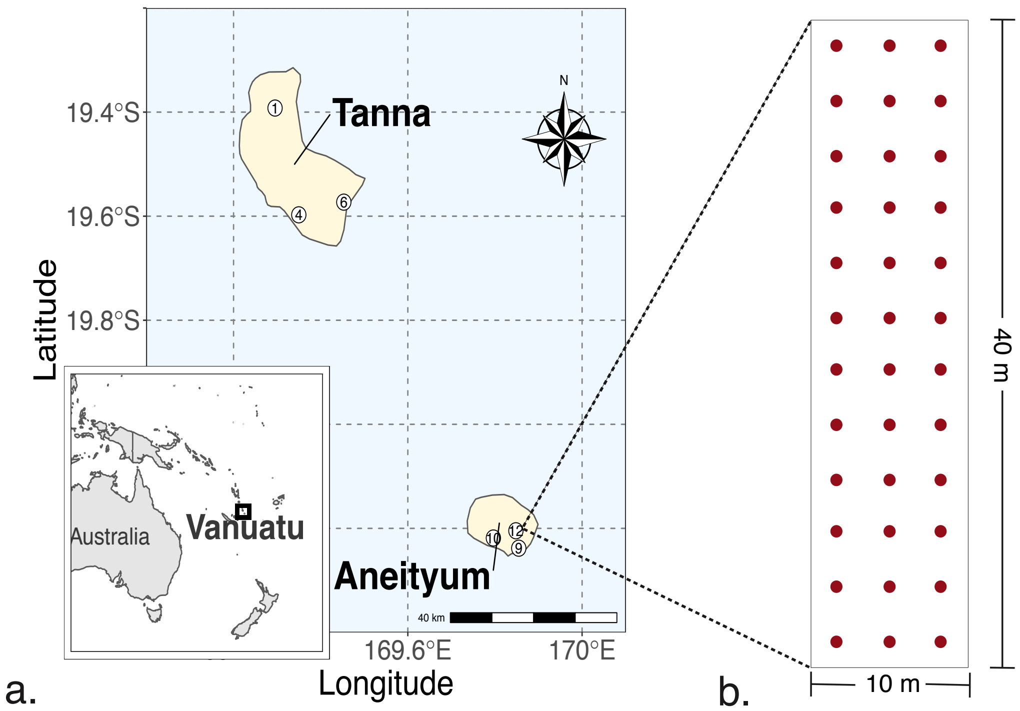

Supplement: Supplemental Information 1 — Red squares on map indicate transect locations (a). Each transect (10 m by 40 m) had 36 sampling locations, indicated by the red circles on the illustrated transect (b). [file peerj-09-11915-s001.pdf]
