# Supplementary material for: Fungi in soil and understory have coupled distribution patterns
Source: PeerJ. 2021 Sep 21;9:e11915. doi: 10.7717/peerj.11915 (PMC8462376; doi:10.7717/peerj.11915)

Fungi in soil and understory have coupled distribution patterns

André Boraks, Anthony S. Amend

PeerJ,2021

Supplemental Figure S2

Flowchart detailing identification of habitat specialist and habitat generalist fungi and the selection process used identify OTUs included in Figure 4

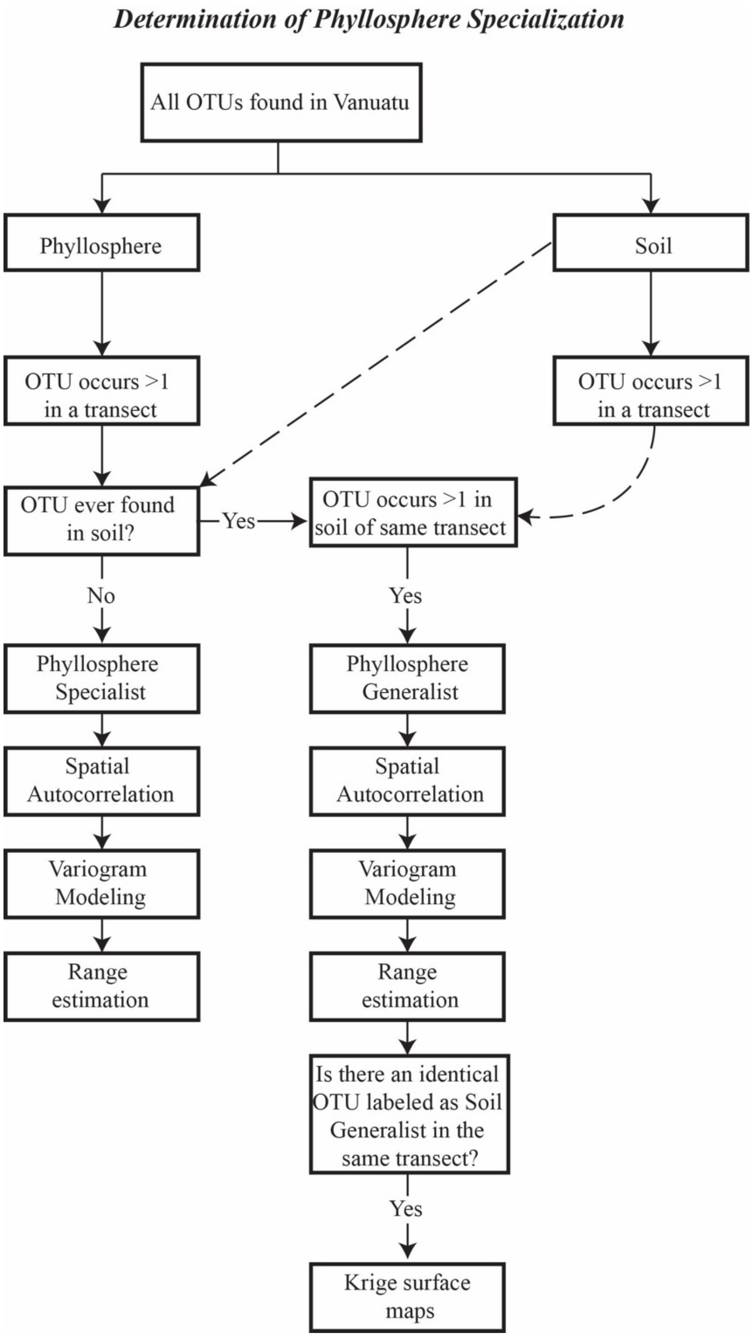

Supplement: Supplemental Information 2 [file peerj-09-11915-s002.pdf]
