# Supplementary material for: Fungi in soil and understory have coupled distribution patterns
Source: PeerJ. 2021 Sep 21;9:e11915. doi: 10.7717/peerj.11915 (PMC8462376; doi:10.7717/peerj.11915)

Soil and leaf fungi have coupled distribution patterns

André Boraks, Anthony S. Amend

PeerJ,2021

Supplemental Figure S3

Generalist OTUs of the same transect were selected (Fig S2) and mapped (Fig 4). This supplemental contains the semi-variogram models, error estimation and kriged surfaces associated with Figure 4.

OTU97_228 Transect 6 Leaf

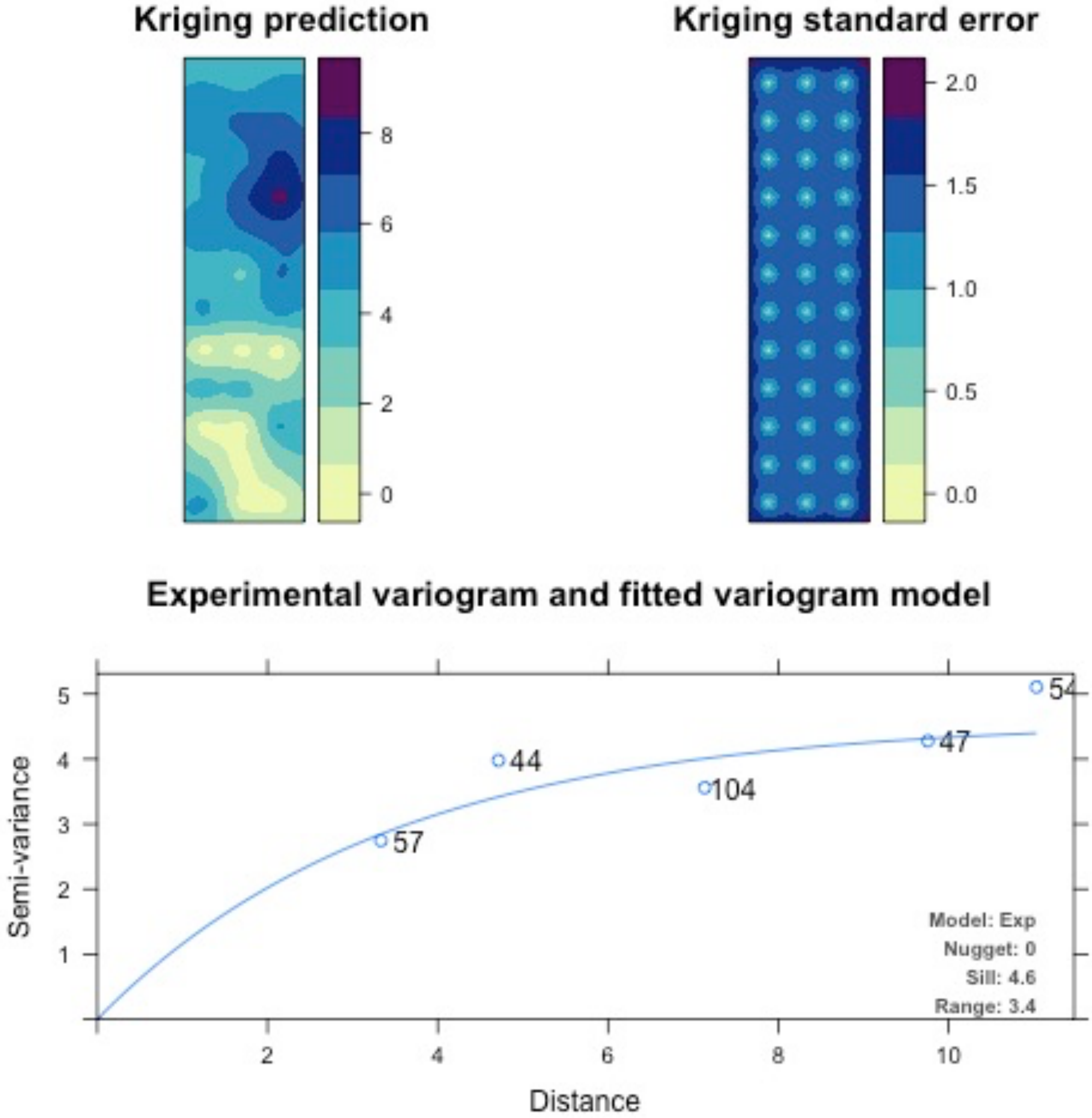

Soil and leaf fungi have coupled distribution patterns
André Boraks, Anthony S. Amend
PeerJ,2021

Supplemental Figure S3
Generalist OTUs of the same transect were selected (Fig S2) and mapped (Fig 4). This supplemental contains the semi-variogram models, error estimation and kriged surfaces associated with Figure 4.

OTU97_228 Transect 6 Soil

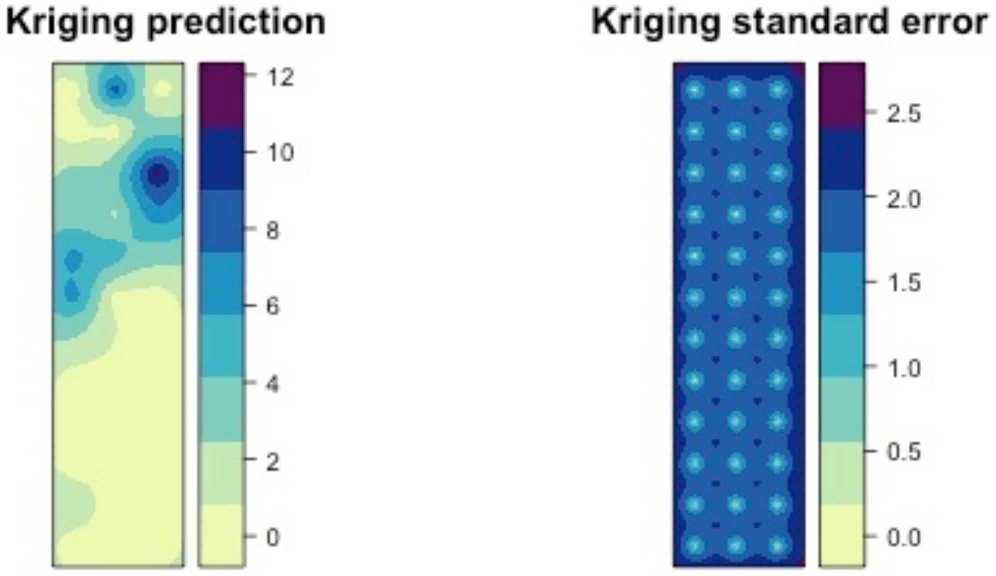

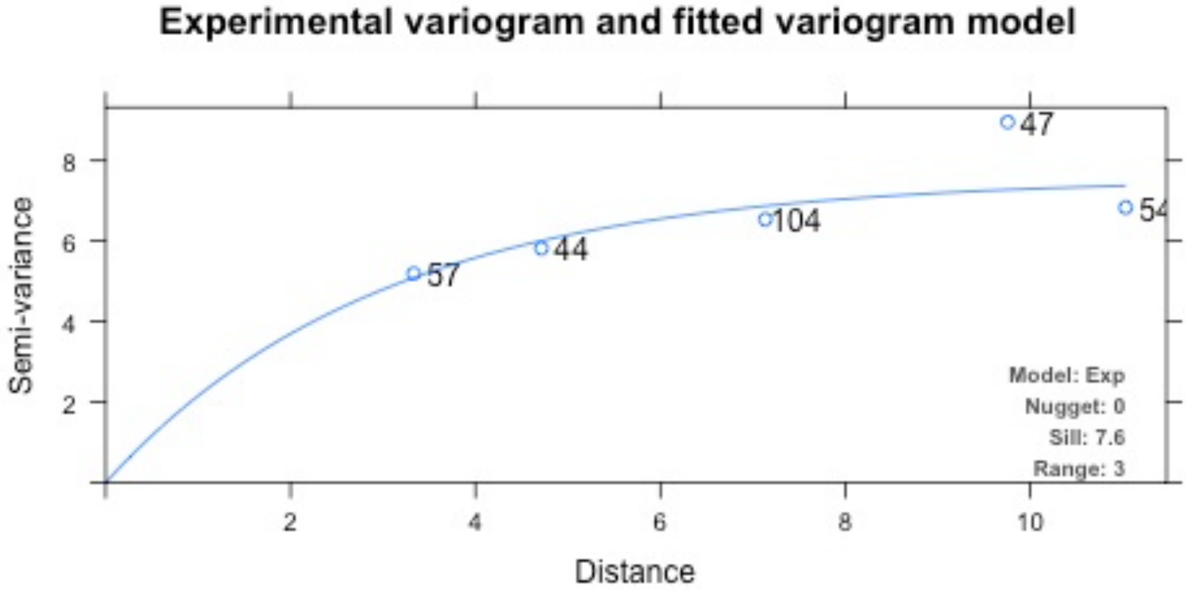

Soil and leaf fungi have coupled distribution patterns
André Boraks, Anthony S. Amend
PeerJ,2021

Supplemental Figure S3

Generalist OTUs of the same transect were selected (Fig S2) and mapped (Fig 4). This supplemental contains the semi-variogram models, error estimation and kriged surfaces associated with Figure 4.

OTU97_257 Transect 12 Leaf

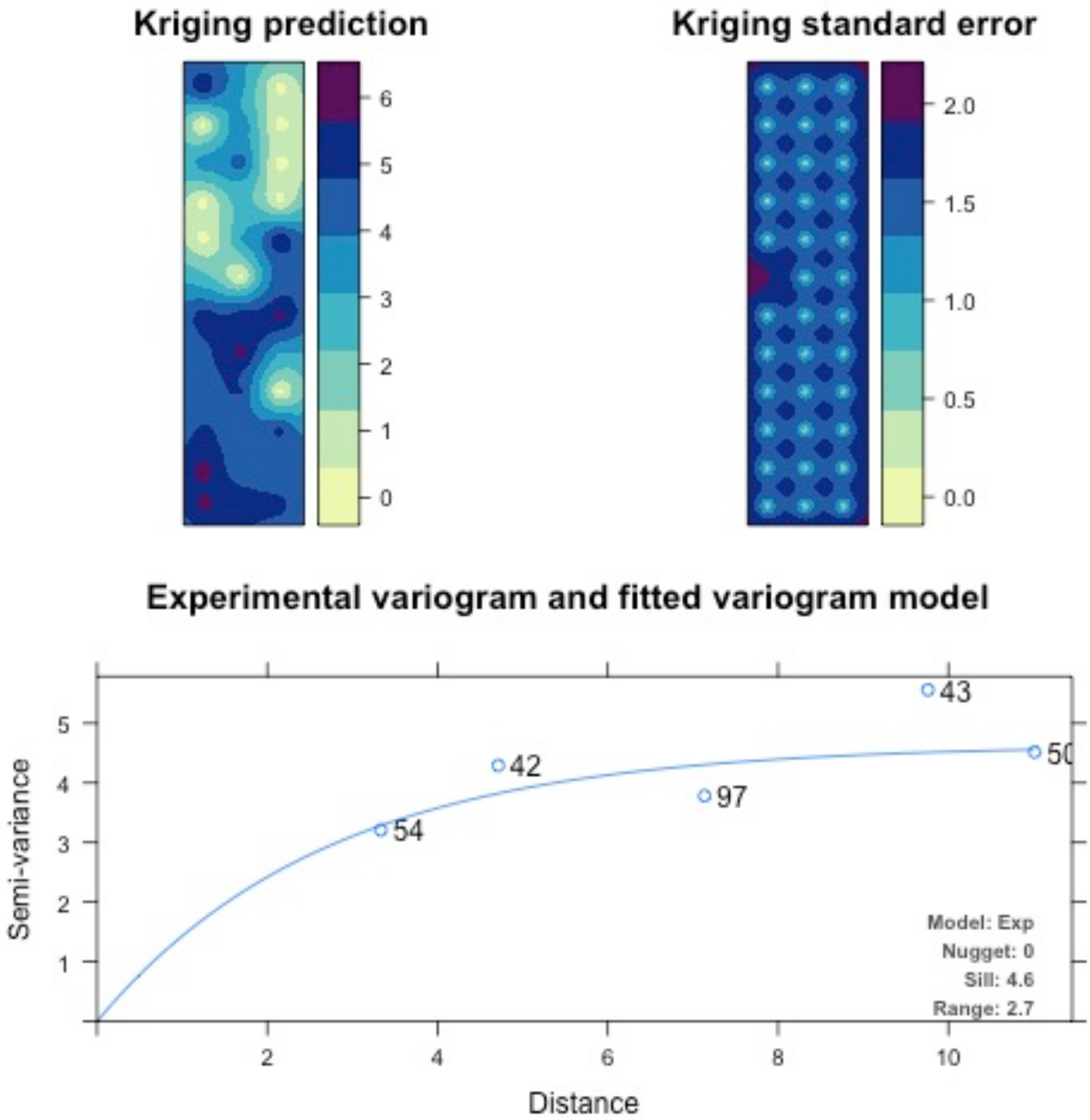

Soil and leaf fungi have coupled distribution patterns
André Boraks, Anthony S. Amend
PeerJ,2021

Supplemental Figure S3

Generalist OTUs of the same transect were selected (Fig S2) and mapped (Fig 4). This supplemental contains the semi-variogram models, error estimation and kriged surfaces associated with Figure 4.

OTU97_257 Transect 12 Soil

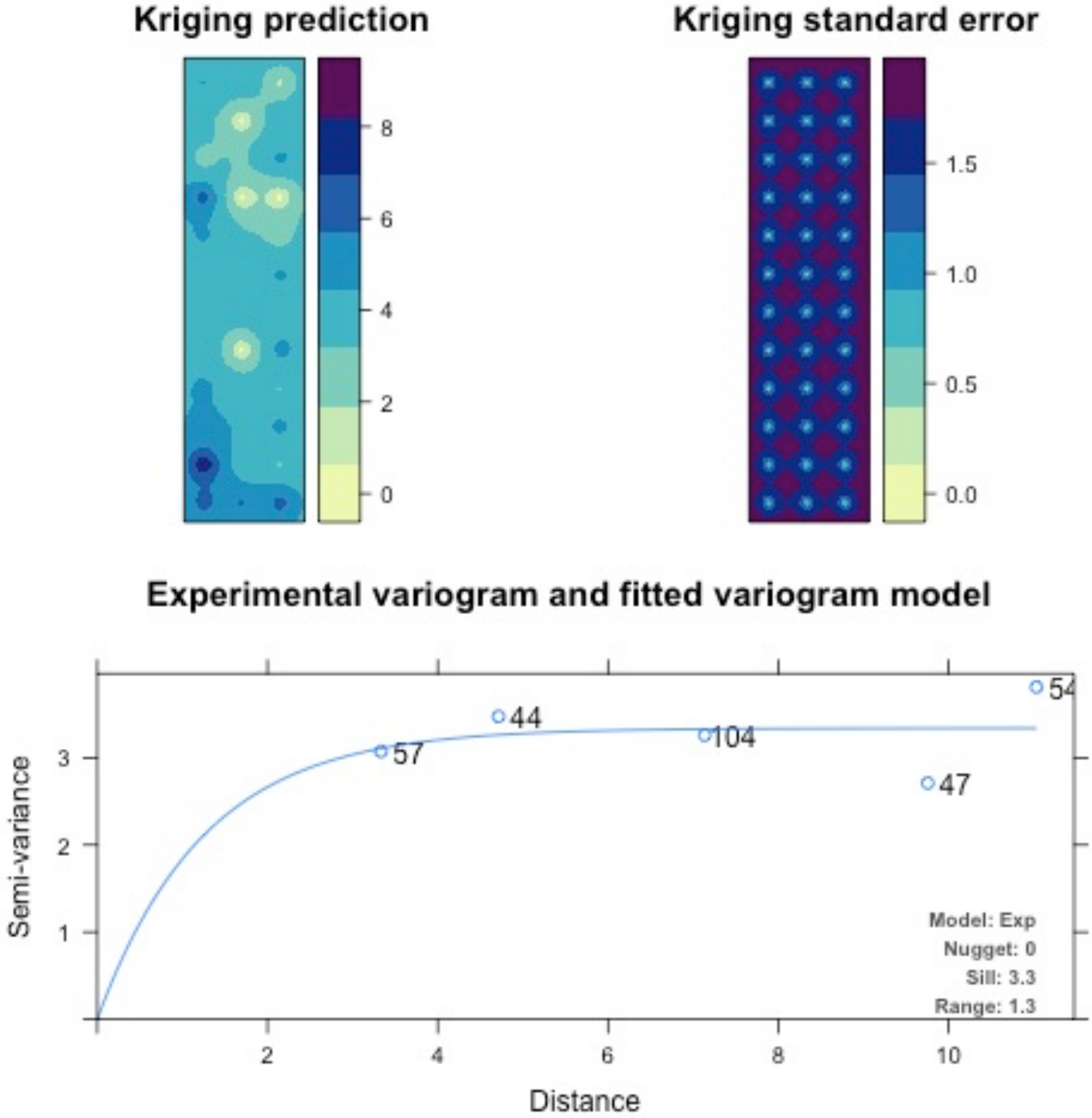

Soil and leaf fungi have coupled distribution patterns
André Boraks, Anthony S. Amend
PeerJ,2021

Supplemental Figure S3

Generalist OTUs of the same transect were selected (Fig S2) and mapped (Fig 4). This supplemental contains the semi-variogram models, error estimation and kriged surfaces associated with Figure 4.

OTU97_1160 Transect 12 Leaf

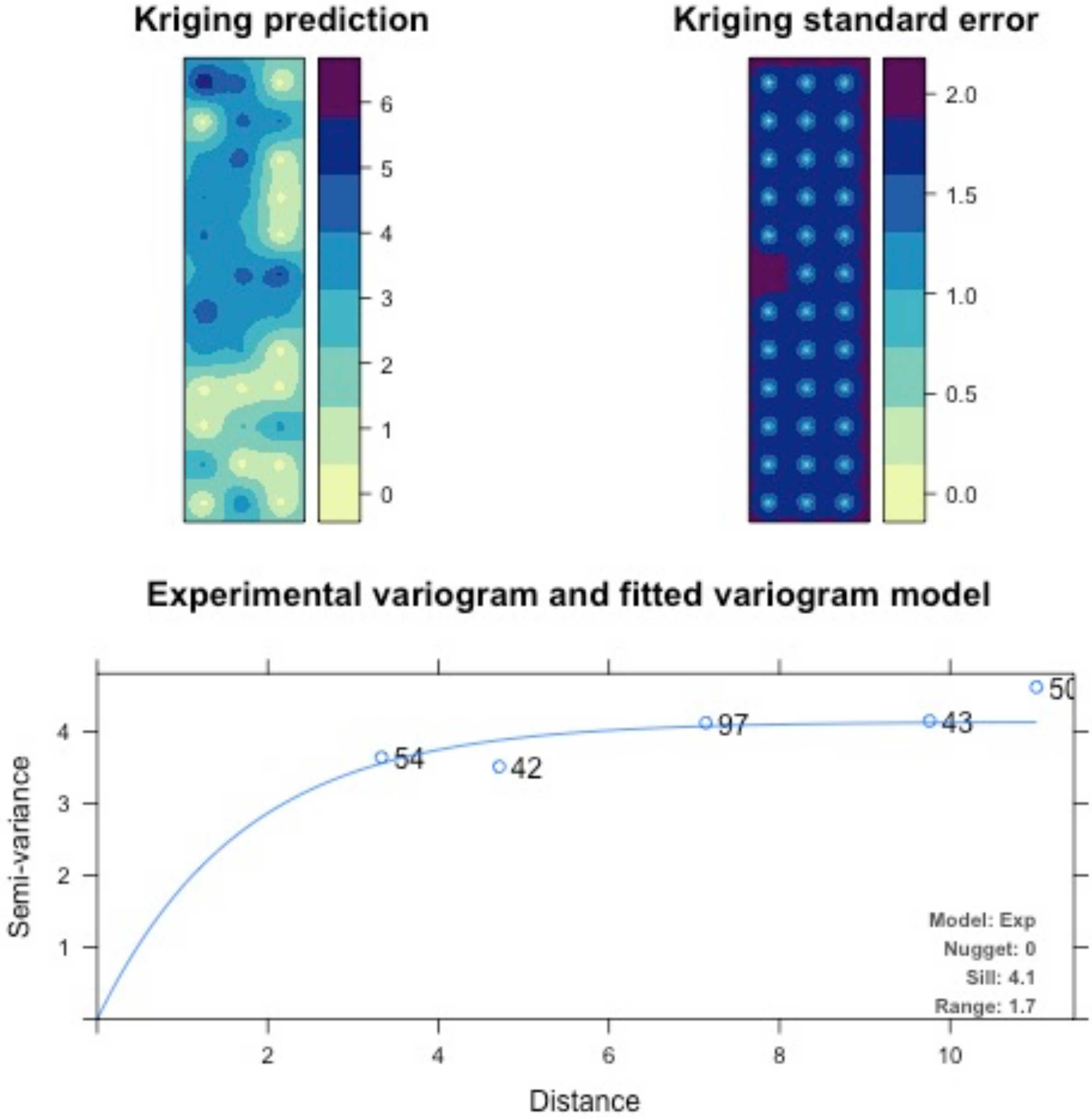

Supplemental Figure S3

Generalist OTUs of the same transect were selected (Fig S2) and mapped (Fig 4). This supplemental contains the semi-variogram models, error estimation and kriged surfaces associated with Figure 4.

OTU97_1160 Transect 12 Soil

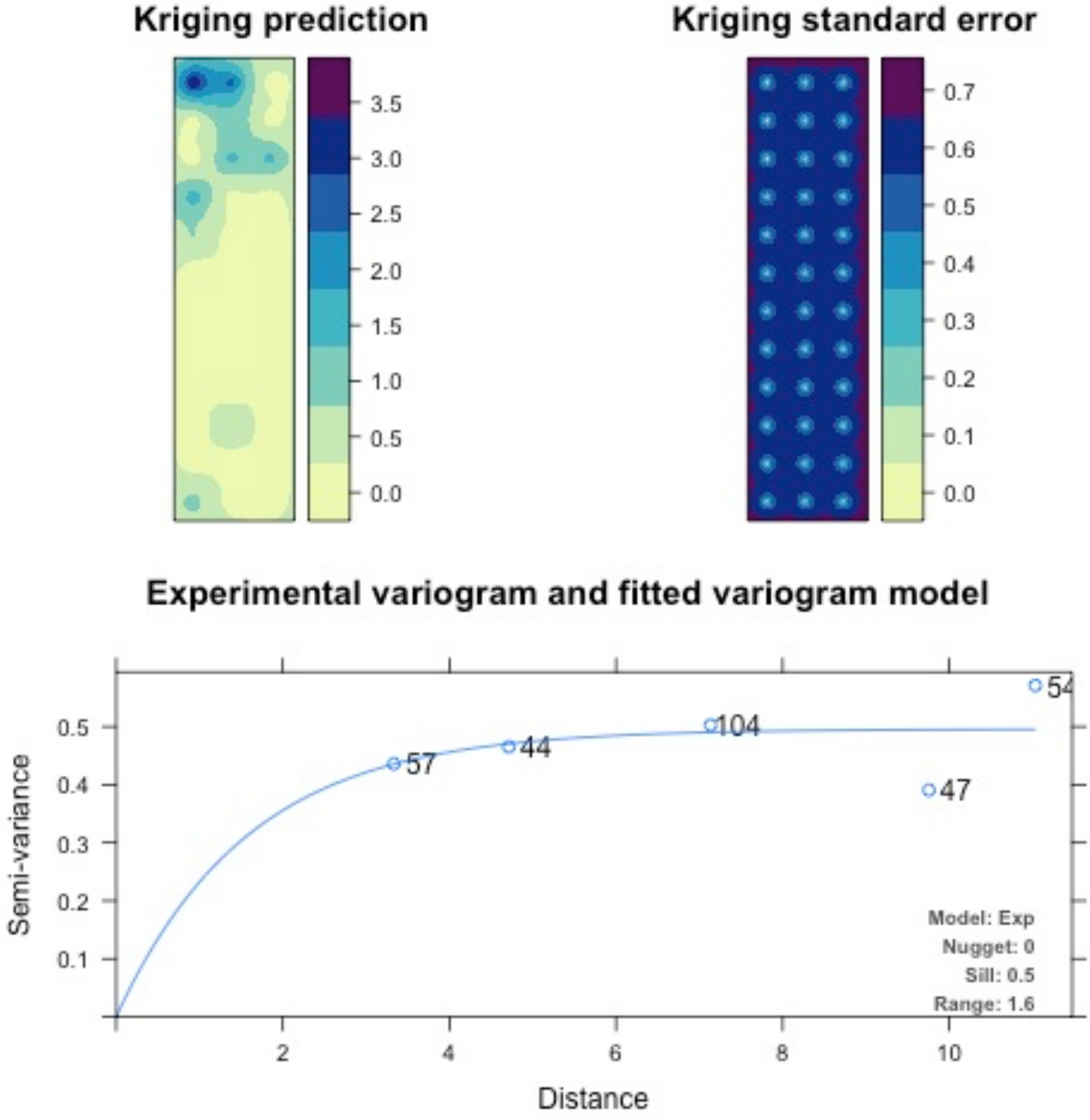

Soil and leaf fungi have coupled distribution patterns
André Boraks, Anthony S. Amend
PeerJ,2021

Supplemental Figure S3

Generalist OTUs of the same transect were selected (Fig S2) and mapped (Fig 4). This supplemental contains the semi-variogram models, error estimation and kriged surfaces associated with Figure 4.

OTU97_1992 Transect 1 Leaf

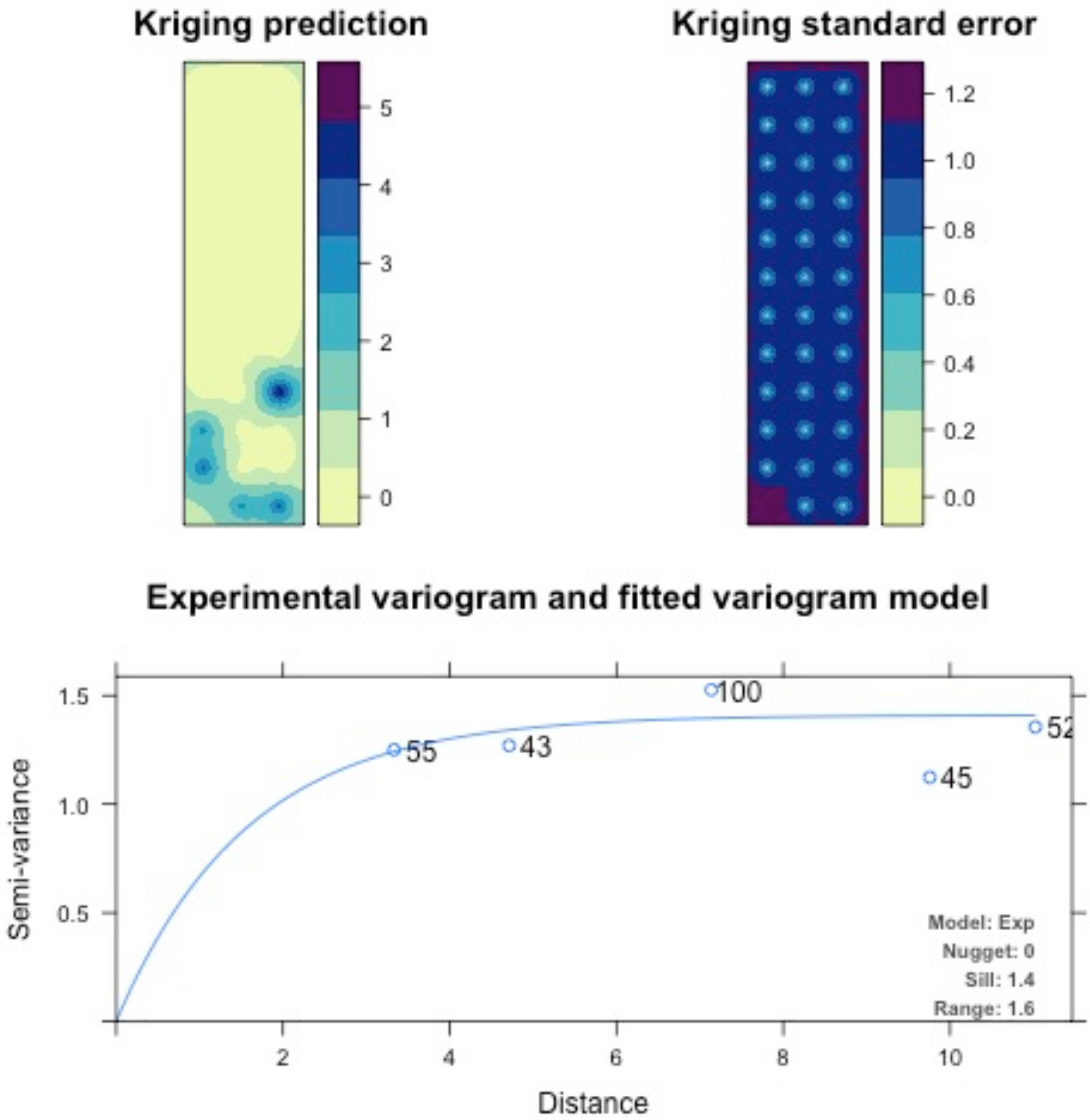

Soil and leaf fungi have coupled distribution patterns
André Boraks, Anthony S. Amend
PeerJ,2021

Supplemental Figure S3

Generalist OTUs of the same transect were selected (Fig S2) and mapped (Fig 4). This supplemental contains the semi-variogram models, error estimation and kriged surfaces associated with Figure 4.

OTU97_1992 Transect 1 Soil

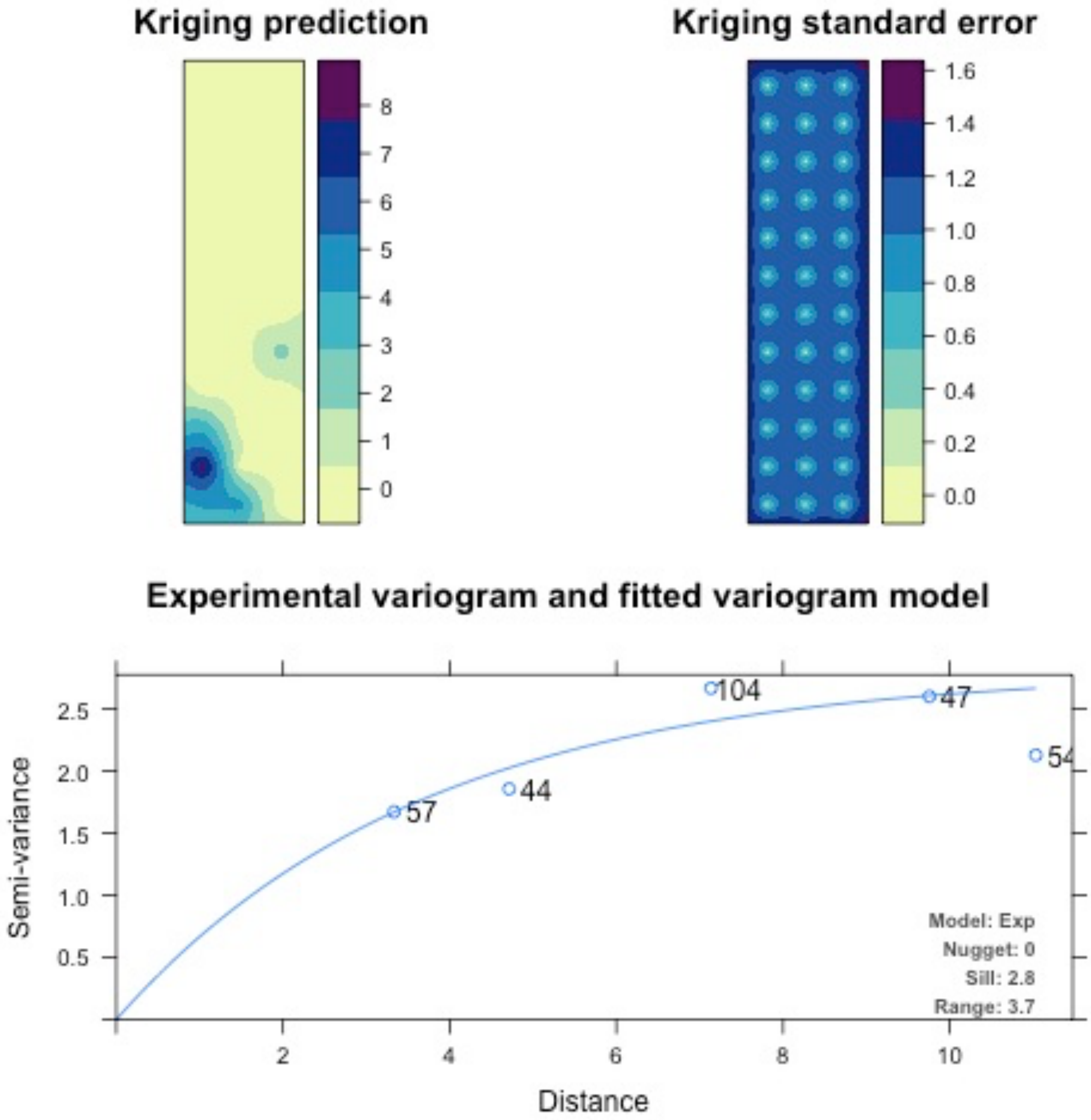

Soil and leaf fungi have coupled distribution patterns
André Boraks, Anthony S. Amend
PeerJ,2021

Supplemental Figure S3

Generalist OTUs of the same transect were selected (Fig S2) and mapped (Fig 4). This supplemental contains the semi-variogram models, error estimation and kriged surfaces associated with Figure 4.

OTU97_2240 Transect 1 Leaf

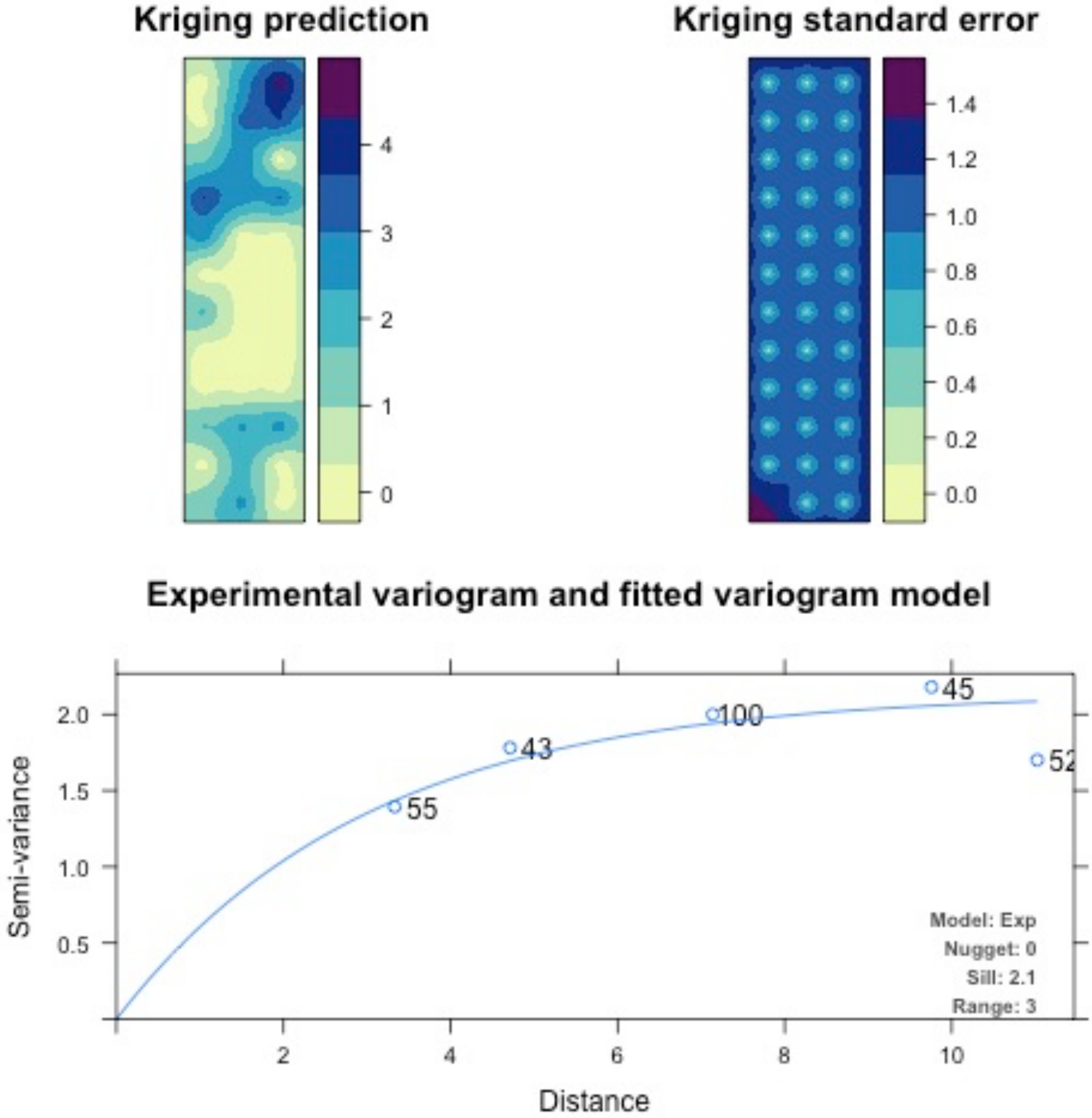

Soil and leaf fungi have coupled distribution patterns
André Boraks, Anthony S. Amend
PeerJ,2021

Supplemental Figure S3

Generalist OTUs of the same transect were selected (Fig S2) and mapped (Fig 4). This supplemental contains the semi-variogram models, error estimation and kriged surfaces associated with Figure 4.

OTU97_2240 Transect 1 Soil

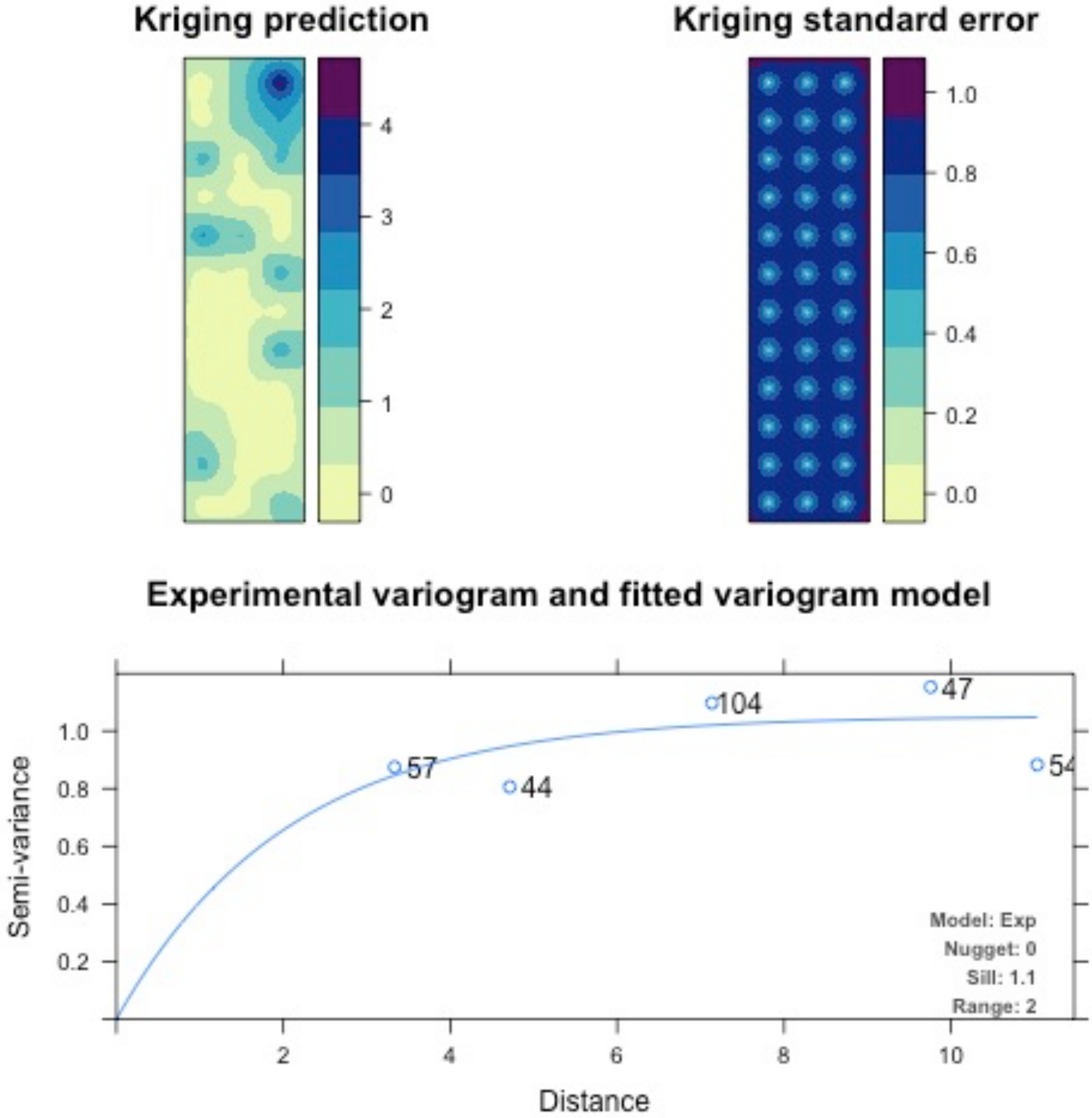

Soil and leaf fungi have coupled distribution patterns
André Boraks, Anthony S. Amend
PeerJ,2021

Supplemental Figure S3

Generalist OTUs of the same transect were selected (Fig S2) and mapped (Fig 4). This supplemental contains the semi-variogram models, error estimation and kriged surfaces associated with Figure 4.

OTU97_2608 Transect 6 Leaf

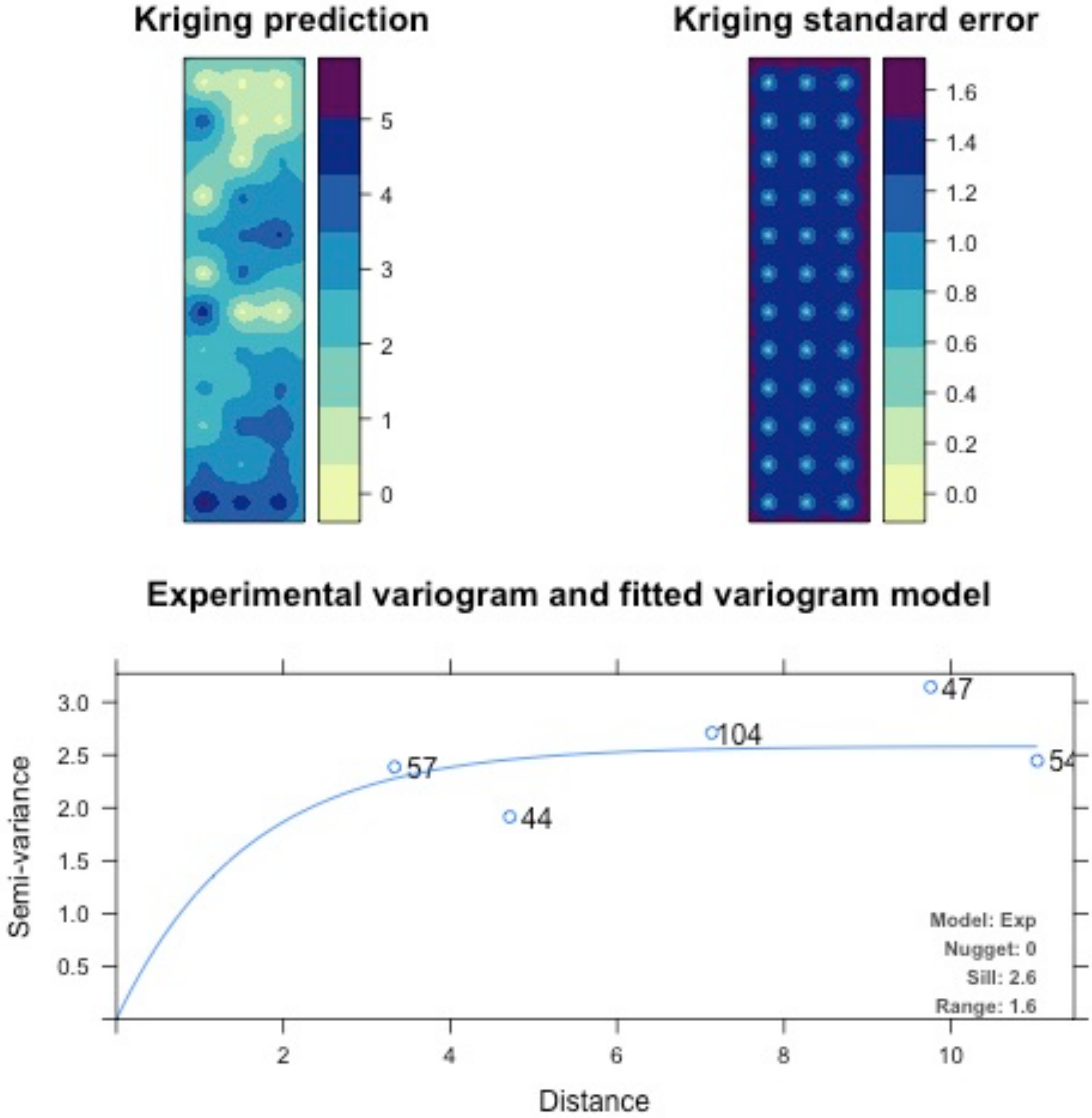

Soil and leaf fungi have coupled distribution patterns
André Boraks, Anthony S. Amend
PeerJ,2021

Supplemental Figure S3

Generalist OTUs of the same transect were selected (Fig S2) and mapped (Fig 4). This supplemental contains the semi-variogram models, error estimation and kriged surfaces associated with Figure 4.

OTU97_2608 Transect 6 Soil

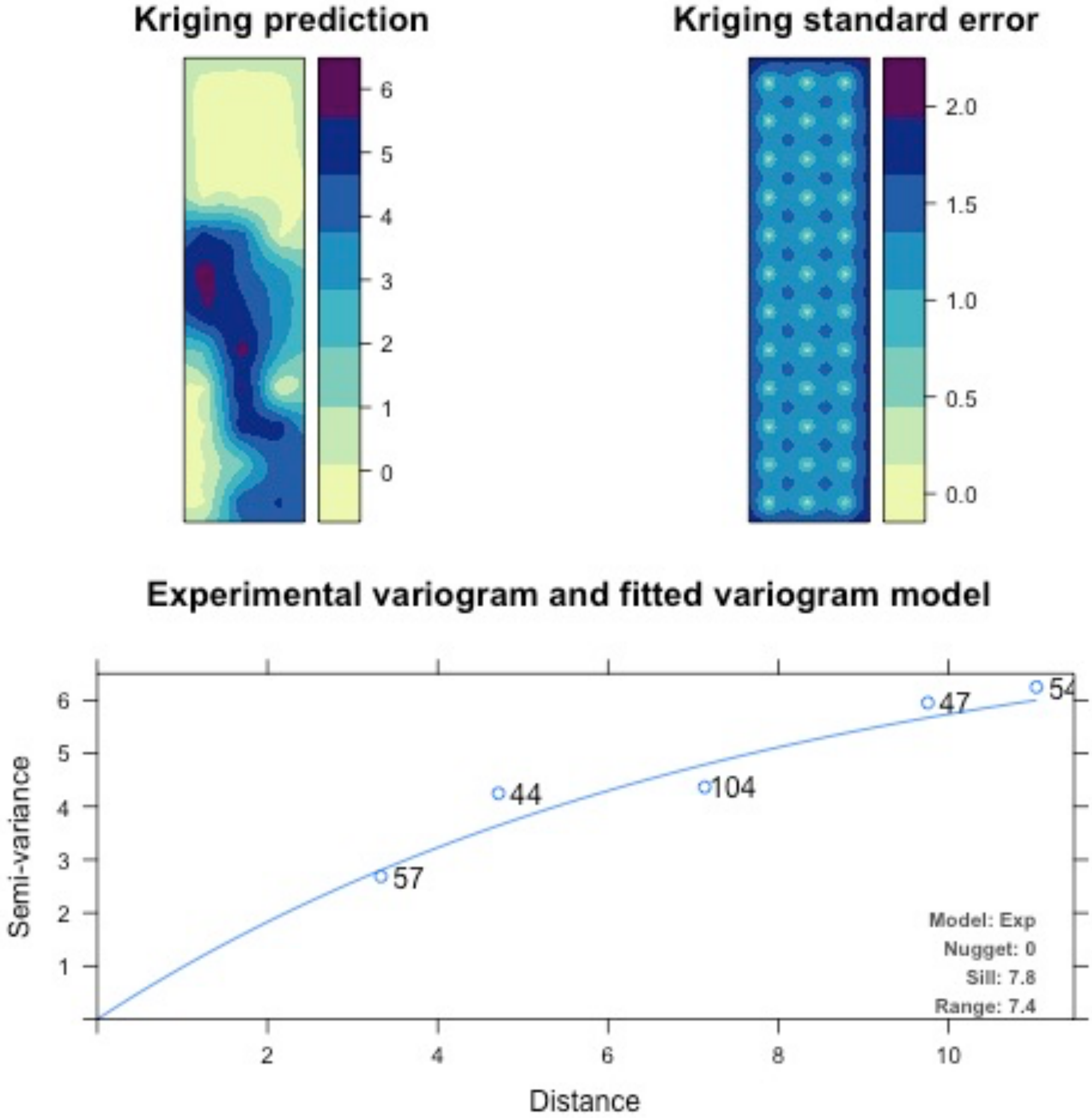

Soil and leaf fungi have coupled distribution patterns
André Boraks, Anthony S. Amend
PeerJ,2021

Supplemental Figure S3

Generalist OTUs of the same transect were selected (Fig S2) and mapped (Fig 4). This supplemental contains the semi-variogram models, error estimation and kriged surfaces associated with Figure 4.

OTU97_2833 Transect 1 Leaf

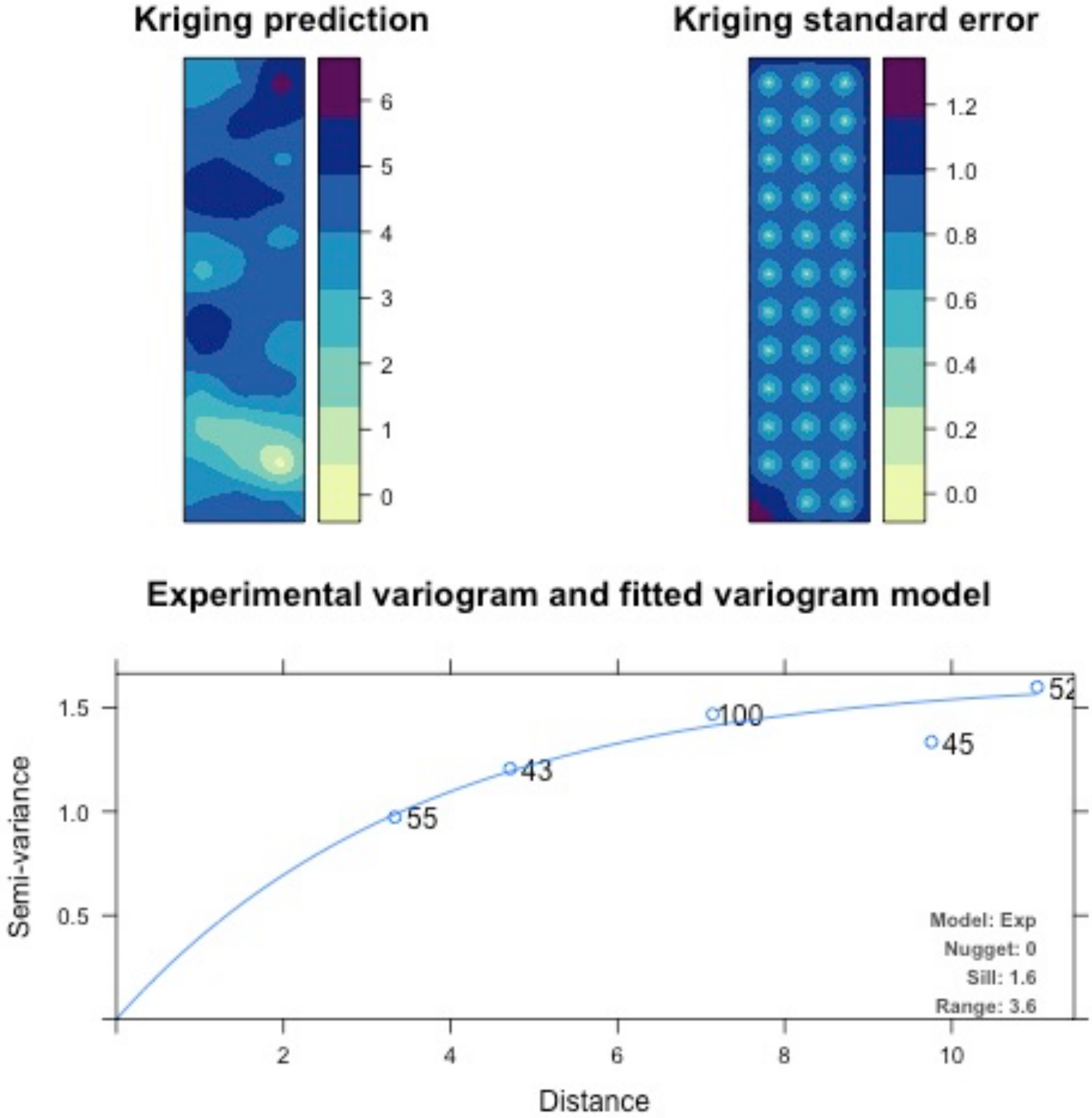

Soil and leaf fungi have coupled distribution patterns
André Boraks, Anthony S. Amend
PeerJ,2021

Supplemental Figure S3

Generalist OTUs of the same transect were selected (Fig S2) and mapped (Fig 4). This supplemental contains the semi-variogram models, error estimation and kriged surfaces associated with Figure 4.

OTU97_2833 Transect 1 Soil

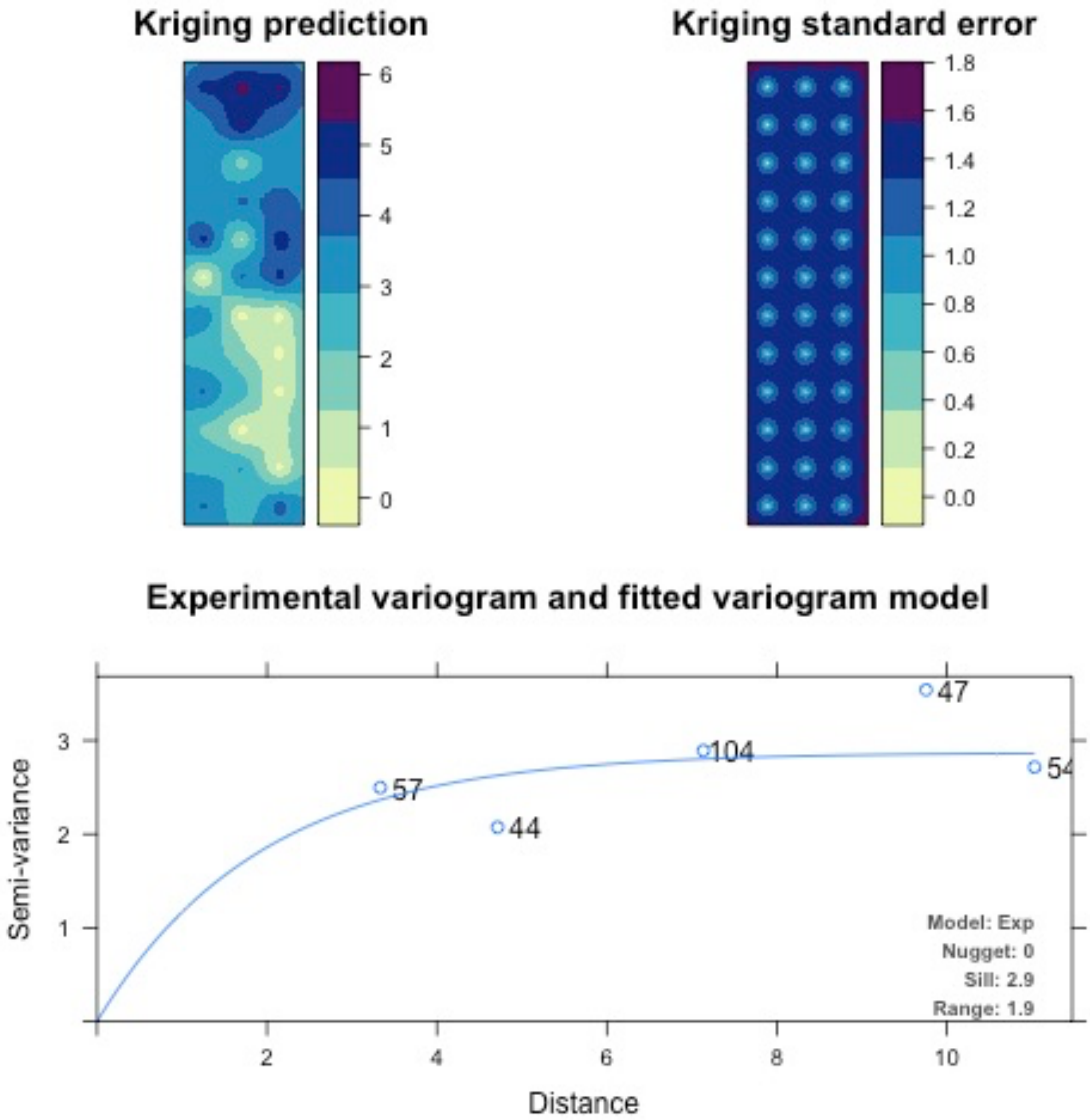

Soil and leaf fungi have coupled distribution patterns
André Boraks, Anthony S. Amend
PeerJ,2021

Supplemental Figure S3

Generalist OTUs of the same transect were selected (Fig S2) and mapped (Fig 4). This supplemental contains the semi-variogram models, error estimation and kriged surfaces associated with Figure 4.

OTU97_3832 Transect 1 Leaf

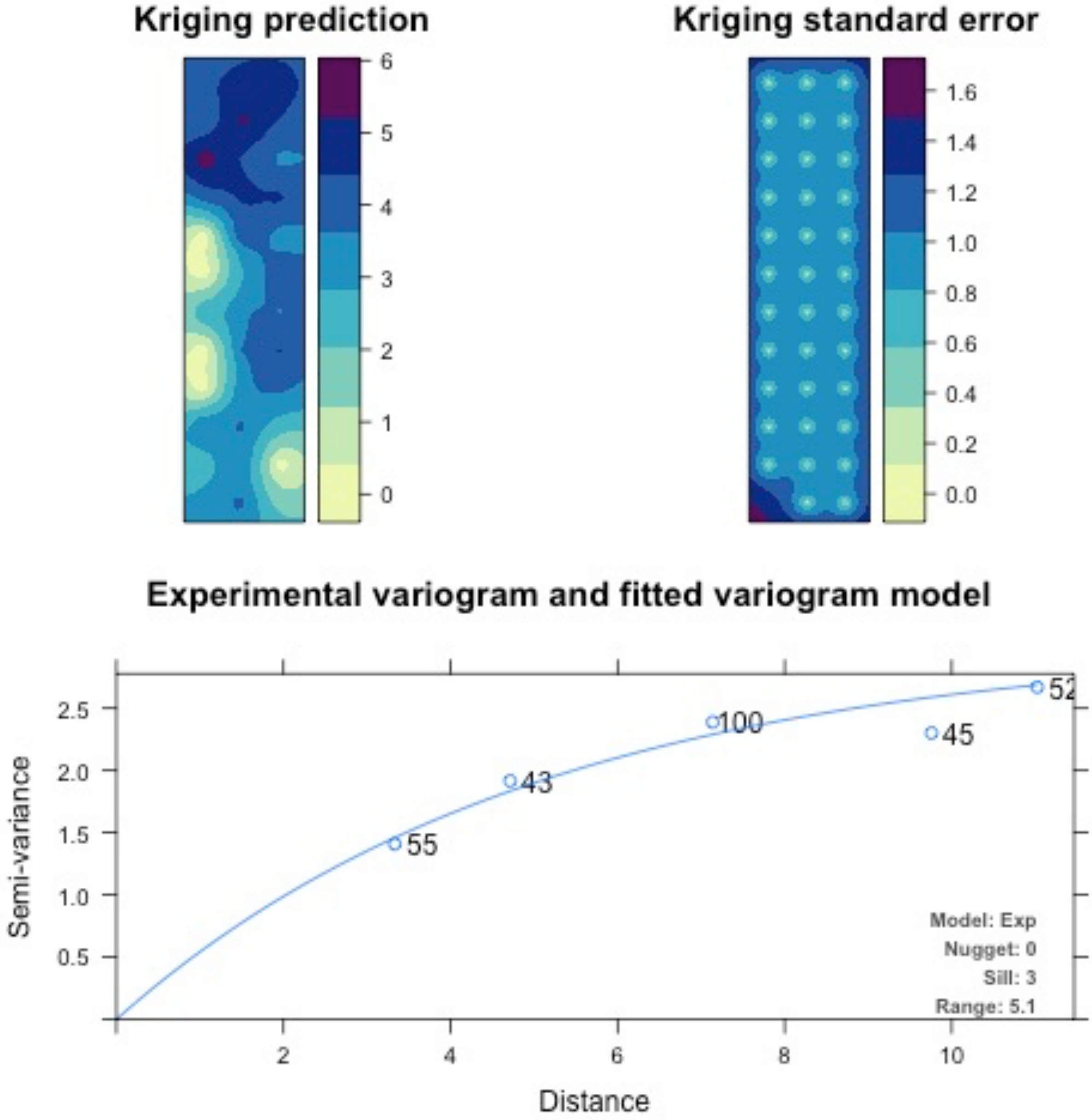

Soil and leaf fungi have coupled distribution patterns
André Boraks, Anthony S. Amend
PeerJ,2021

Supplemental Figure S3
Generalist OTUs of the same transect were selected (Fig S2) and mapped (Fig 4). This supplemental contains the semi-variogram models, error estimation and kriged surfaces associated with Figure 4.

OTU97_3832 Transect 1 Soil

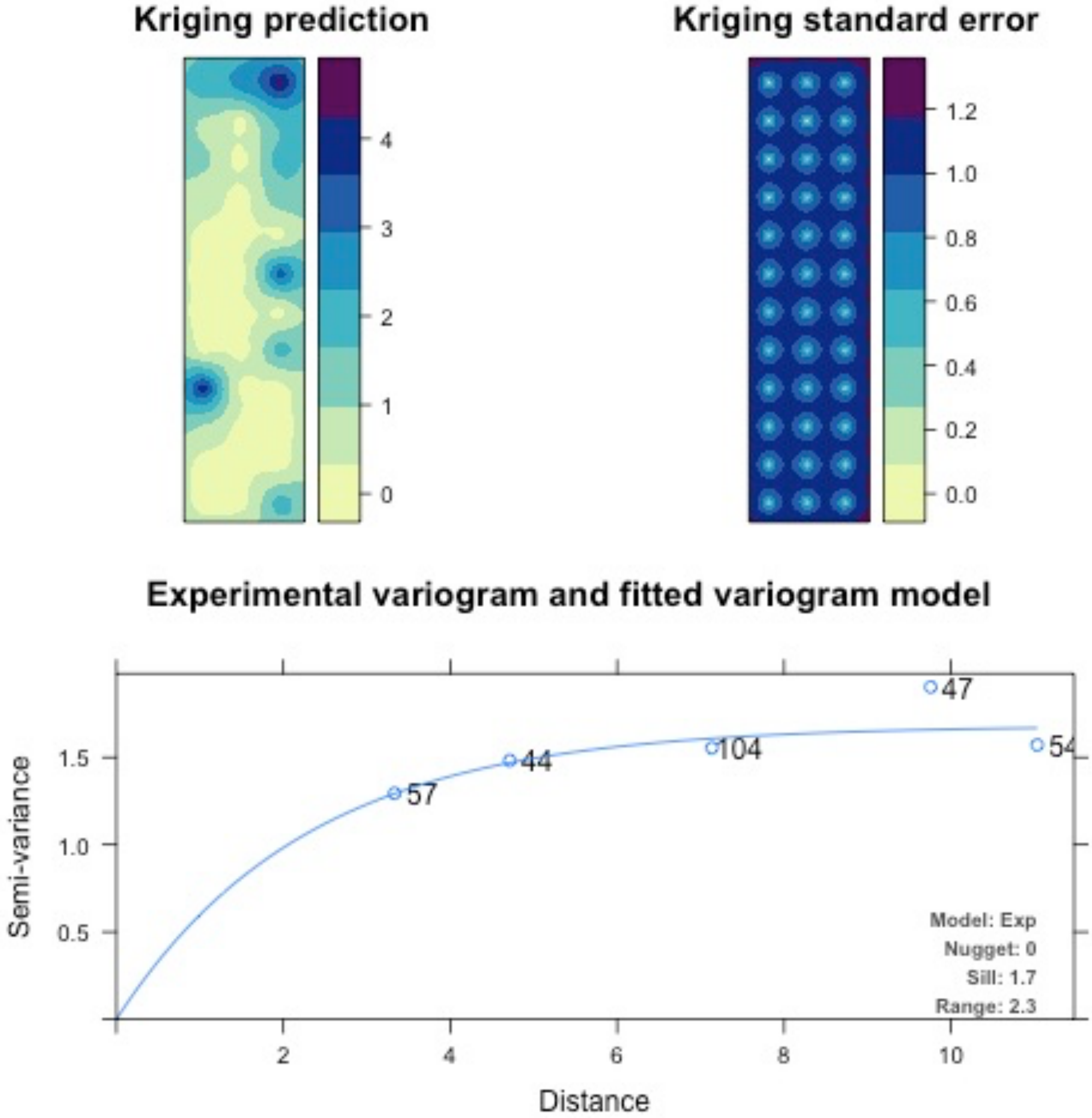

Soil and leaf fungi have coupled distribution patterns
André Boraks, Anthony S. Amend
PeerJ,2021

Supplemental Figure S3

Generalist OTUs of the same transect were selected (Fig S2) and mapped (Fig 4). This supplemental contains the semi-variogram models, error estimation and kriged surfaces associated with Figure 4.

OTU97_3832 Transect 6 Leaf

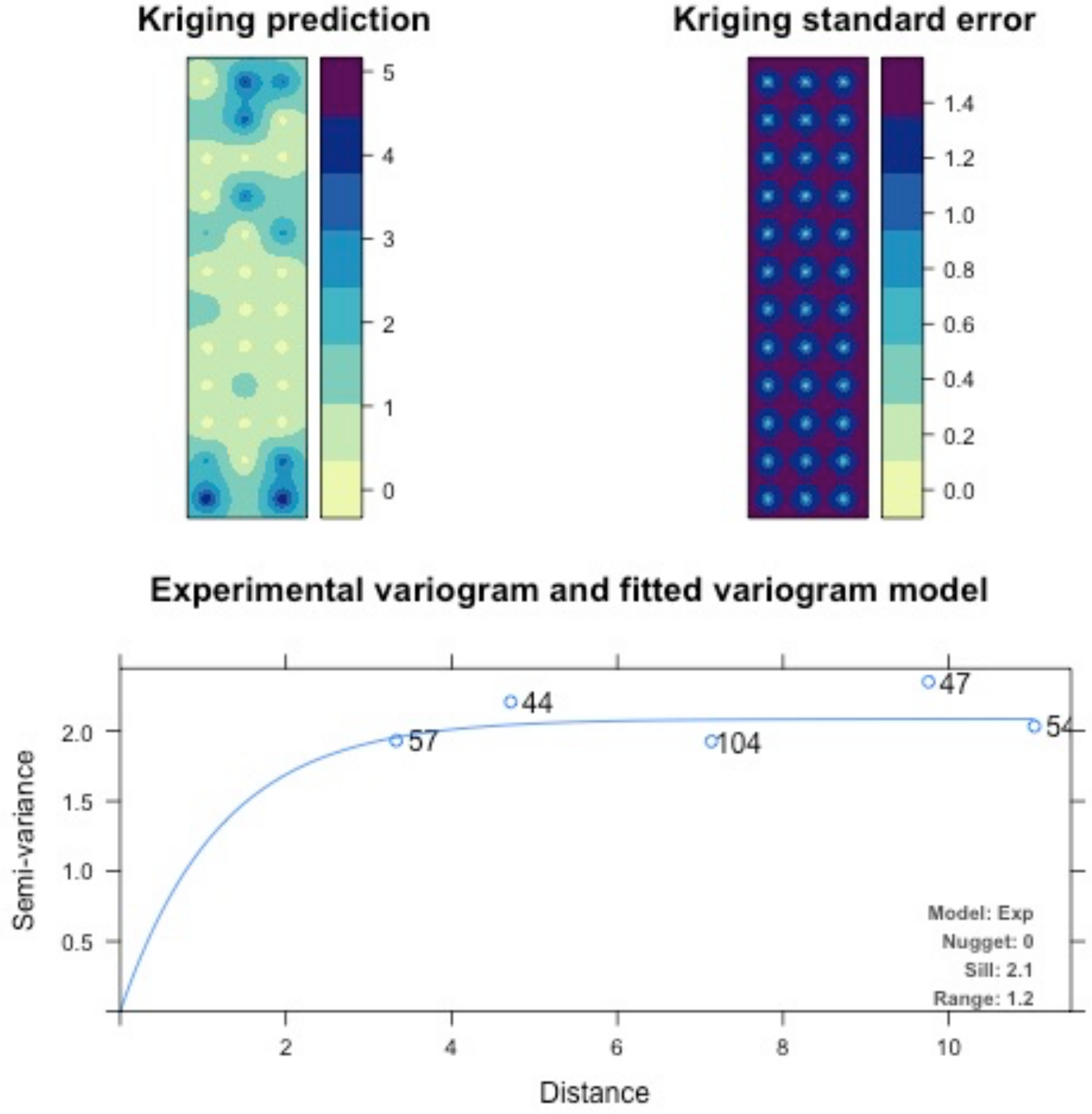

Soil and leaf fungi have coupled distribution patterns
André Boraks, Anthony S. Amend
PeerJ,2021

Supplemental Figure S3

Generalist OTUs of the same transect were selected (Fig S2) and mapped (Fig 4). This supplemental contains the semi-variogram models, error estimation and kriged surfaces associated with Figure 4.

OTU97_3832 Transect 6 Soil

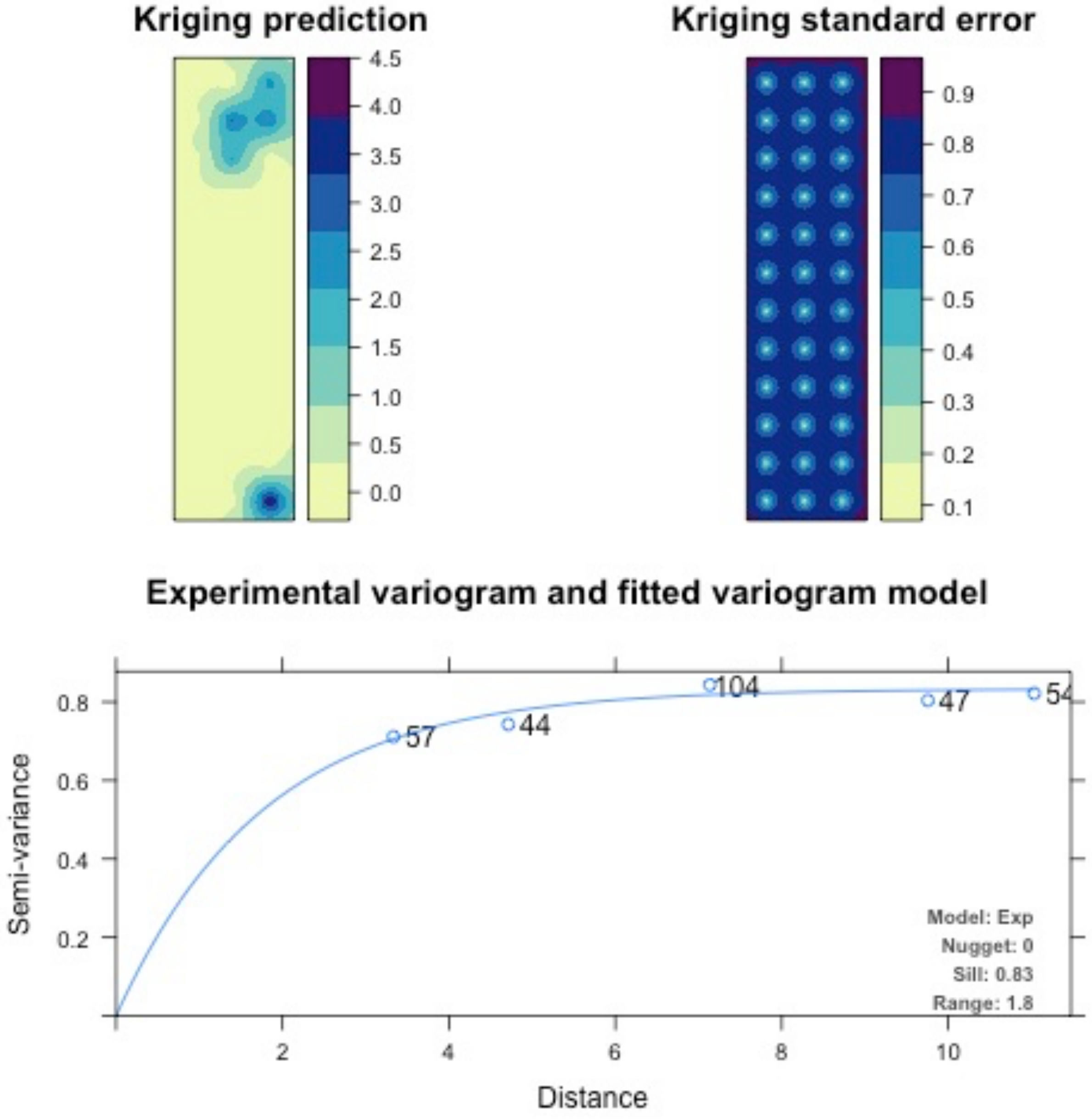

Soil and leaf fungi have coupled distribution patterns
André Boraks, Anthony S. Amend
PeerJ,2021

Supplemental Figure S3

Generalist OTUs of the same transect were selected (Fig S2) and mapped (Fig 4). This supplemental contains the semi-variogram models, error estimation and kriged surfaces associated with Figure 4.

OTU97_5320 Transect 10 Leaf

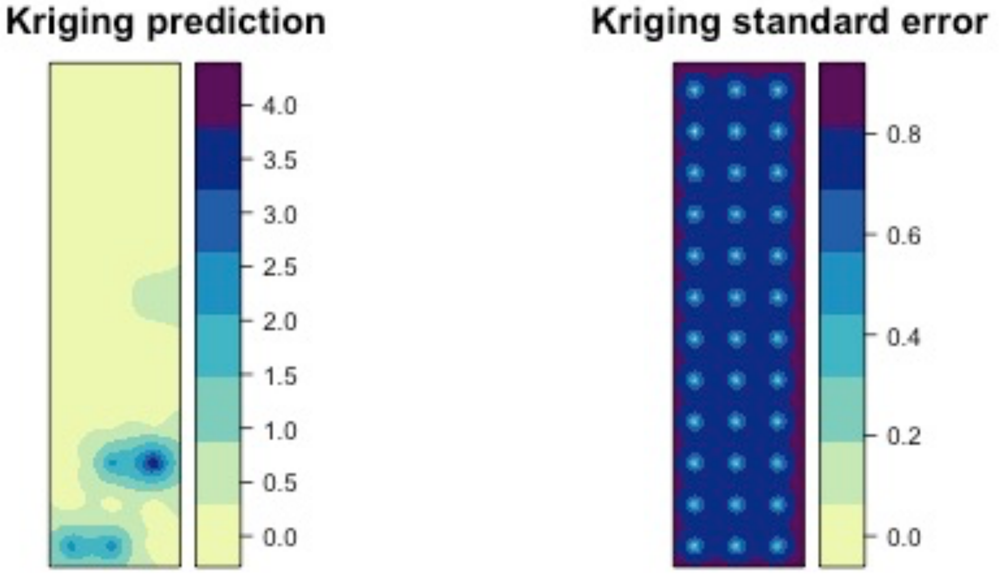

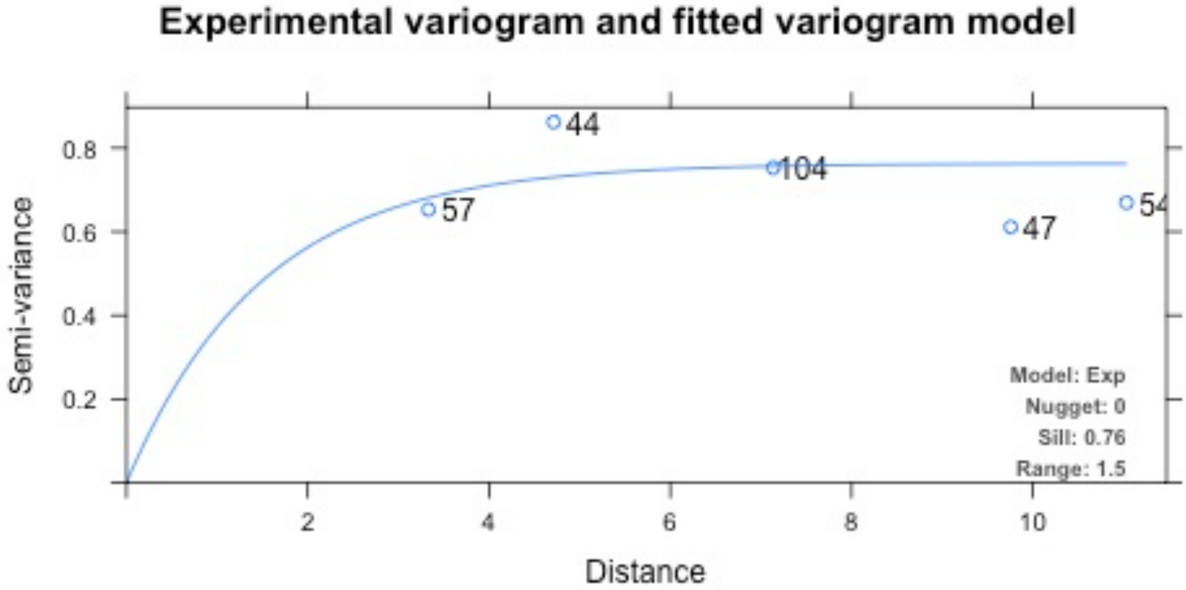

Soil and leaf fungi have coupled distribution patterns
André Boraks, Anthony S. Amend
PeerJ,2021

Supplemental Figure S3

Generalist OTUs of the same transect were selected (Fig S2) and mapped (Fig 4). This supplemental contains the semi-variogram models, error estimation and kriged surfaces associated with Figure 4.

OTU97_5320 Transect 10 Soil

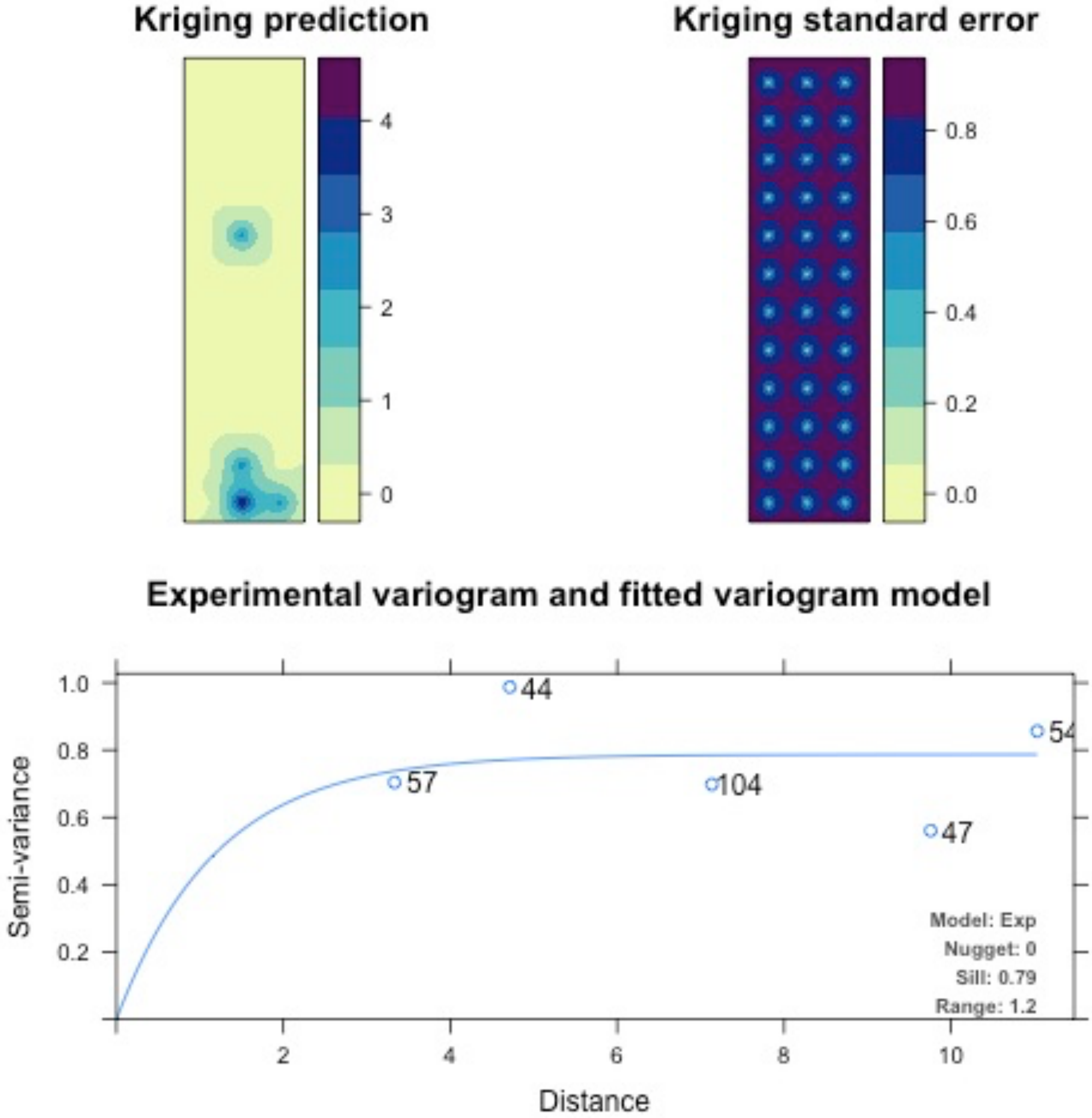

Soil and leaf fungi have coupled distribution patterns
André Boraks, Anthony S. Amend
PeerJ,2021

Supplemental Figure S3

Generalist OTUs of the same transect were selected (Fig S2) and mapped (Fig 4). This supplemental contains the semi-variogram models, error estimation and kriged surfaces associated with Figure 4.

OTU97_6890 Transect 6 Leaf

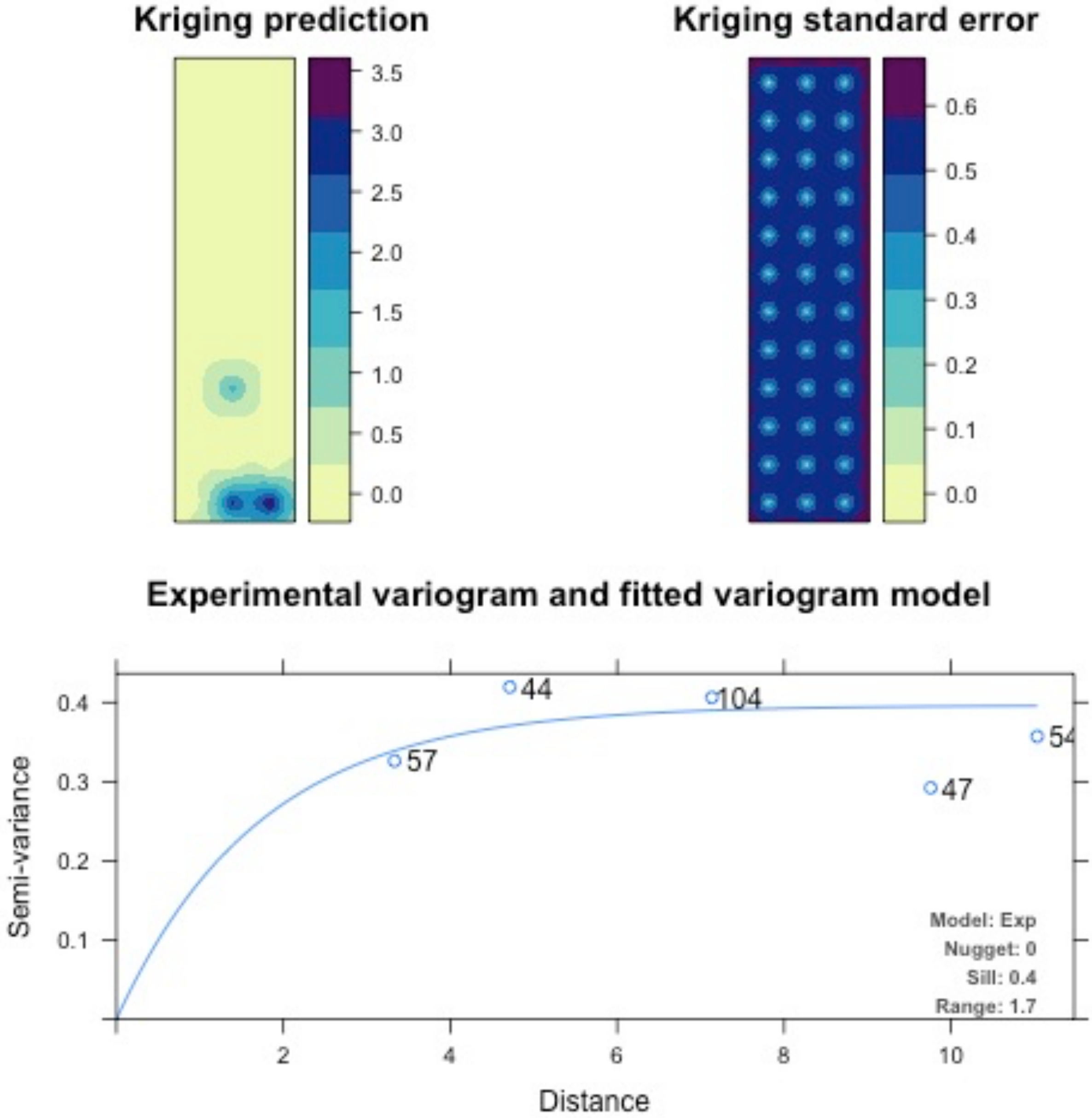

Soil and leaf fungi have coupled distribution patterns
André Boraks, Anthony S. Amend
PeerJ,2021

Supplemental Figure S3

Generalist OTUs of the same transect were selected (Fig S2) and mapped (Fig 4). This supplemental contains the semi-variogram models, error estimation and kriged surfaces associated with Figure 4.

OTU97_6890 Transect 6 Soil

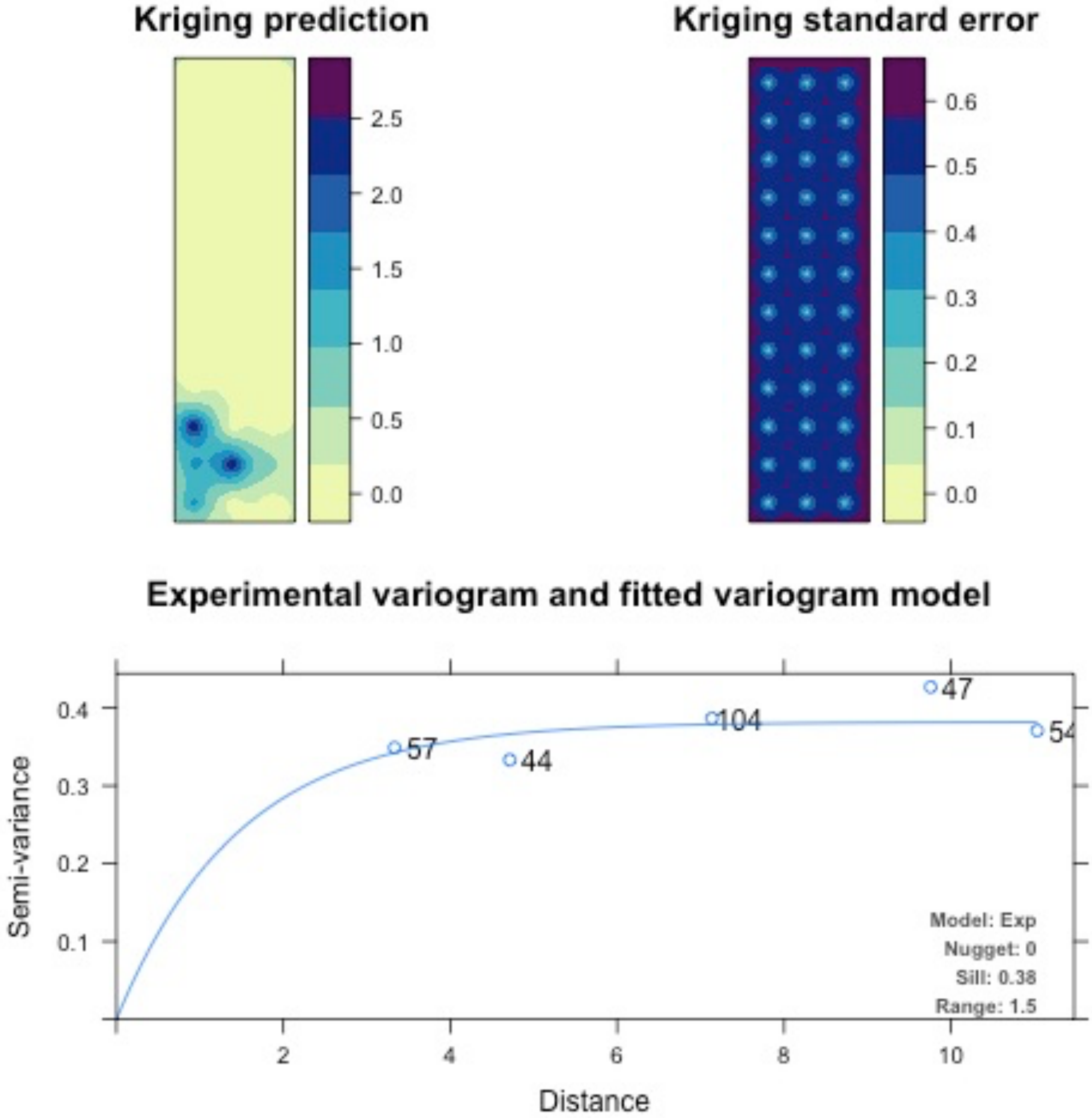

Supplement: Supplemental Information 3 — Generalist OTUs of the same transect were selected by protocol (Fig. S2) and mapped using kriging. This supplemental contains a compilation of the semi-variogram models, error estimation, and kriged surfaces associated with maps published as Fig. 4. [file peerj-09-11915-s003.pdf]
